# Prognóstico Relacionado à Terapia de Reperfusão Pós-Síndrome Coronariana Aguda na Atenção Secundária: Análise de Sobrevida em Longo Prazo na Estratégia ERICO

**DOI:** 10.36660/abc.20220849

**Published:** 2023-05-02

**Authors:** Tatiana C. Bruno, Márcio S. Bittencourt, Alessandra V. L. Quidim, Itamar S. Santos, Paulo A. Lotufo, Isabela M. Benseñor, Alessandra C. Goulart

**Affiliations:** 1 Centro de Pesquisa Clínica e Epidemiológica Hospital Universitário Universidade de São Paulo São Paulo SP Brasil Centro de Pesquisa Clínica e Epidemiológica – Hospital Universitário – Universidade de São Paulo , São Paulo , SP – Brasil; 2 Universidade de São Paulo Faculdade de Medicina São Paulo SP Brasil Universidade de São Paulo , Faculdade de Medicina , São Paulo , SP – Brasil

**Keywords:** Síndrome Coronariana Aguda, Mortalidade, Análise de Sobrevida, Angioplastia, Revascularização do Miocárdio

## Abstract

**Fundamento:**

A relação entre terapia de reperfusão após a síndrome coronariana aguda (SCA) e mortalidade na atenção secundária não é bem conhecida.

**Objetivos:**

Avaliar o impacto de três estratégias terapêuticas: (1) terapia medicamentosa exclusiva, (2) Angioplastia Transluminal percutânea coronaria (ATPC) e (3) revascularização do miocárdio (RM) na sobrevida em longo prazo de participantes da Estratégia de Registro de Insuficiência Coronariana Aguda (ERICO).

**Métodos:**

Análises de sobrevida para mortalidade por todas as causas, mortalidade por doença cardiovascular (DCV) e mortalidade por doença arterial coronariana (DAC) foram realizadas de acordo com três estratégias terapêuticas (tratamento clínico exclusivo, ATPC ou RM). Modelos de regressão de Cox foram usados para estimar o hazard ratio (HR) com intervalo de confiança de 95% (IC95%) de 180 dias a quatro anos de acompanhamento após a SCA. Os modelos são apresentados como modelo sem ajuste ou ajustado quanto à idade, sexo e DAC prévia, tipo de SCA, tabagismo, hipertensão, dislipidemia, fração de ejeção do ventrículo esquerdo e de acordo com o número de artérias coronárias principais obstruídas (≥50%).

**Resultados:**

Entre os 800 participantes, as piores taxas de sobrevida (mortalidade por todas as causas e DCV) foram detectadas entre os indivíduos que se submeteram a RM. Houve correlação entre RM e DAC [HR: 2,19 (IC95% 1,05-4,55)], mas o risco perdeu significância no modelo multivariado. A ATPC foi associada a uma menor probabilidade de eventos fatais durante os quatro anos de acompanhamento: mortalidade por todas as causas [HR, análise multivariada: 0,42 (IC95% 0,26-0,70)], por DCV [HR: 0,39 (95% CI: 0,20-0,73)] e DAC [HR, análise multivariada: 0,24 (IC95% 0,09-0,63)] em comparação aos submetidos ao tratamento clínico exclusivo.

**Conclusão:**

No ERICO, a ATPC após a SCA foi associada a um melhor prognóstico, principalmente sobrevida por DAC.

## Introdução

A doença cardiovascular (DCV) é causa de altas taxas de mortalidade, principalmente em países em desenvolvimento. ^1 , 2^ No Brasil, a DCV foi a principal causa de morte nas últimas décadas, sendo a doença arterial coronariana (DAC) responsável por um terço das mortes por DCV em 2016. ^3^

Apesar dos avanços no tratamento cardiovascular com técnicas cirúrgicas e surgimento dos *stents* farmacológicos, ^4^ estudos recentes que compararam diferentes estratégias terapêuticas após síndrome coronariana aguda (SCA), como angioplastia Transluminal percutânea coronaria (ATPC), revascularização do miocárdio (RM), e terapia medicamentosa exclusiva relataram achados controversos quanto ao impacto sobre a mortalidade. ^5 - 8^ Em países em desenvolvimento, a desigualdade socioeconômica associada à falta de acesso a centros terciários de cardiologia representa uma barreira a tratamentos mais efetivos durante a fase aguda de um evento coronário. O tratamento inadequado tem contribuído para as altas taxas de mortalidade no Brasil nas últimas décadas. ^9^ Assim, nós avaliamos o prognóstico em longo prazo de pacientes com SCA, participantes do ERICO (Estratégia de Registro de Insuficiência Coronariana Aguda) de acordo com o tratamento implementado após a SCA. Comparamos três estratégias de tratamento: (1) terapia medicamentosa exclusiva, (2) ATPC (angioplastia com balão, *stents* farmacológicos, e *stents* de metal), e (3) RM.

## Métodos

### População e delineamento do estudo

Todos os pacientes eram participantes do ERICO, um estudo prospectivo do tipo coorte de paciente com SCA recrutados no Hospital Universitário da Universidade de São Paulo (HU-USP). ^10^

Em resumo, o ERICO é um estudo em andamento no HU-USP, um hospital secundário com 260 leitos localizado no bairro do Butantã, São Paulo, cuja população era de 428 mil habitantes em 2010. ^9 , 11^ Embora o Butantã apresente alguns indicadores socioeconômicos acima da média da cidade de São Paulo (por exemplo, da renda familiar), ele é caracterizado por amplas desigualdades. ^11^ Os participantes do ERICO residem em uma região de renda média-baixa, com possíveis dificuldades de acesso aos serviços de saúde.

Neste estudo, avaliamos todos os participantes (n=800/1085, 73,7%) admitidos no departamento de emergência do HU-USP, com SCA confirmada, submetidos à angiografia invasiva para o diagnóstico de obstrução coronariana, seguido de tratamento após a fase aguda com tratamento clínico isolado, incluindo trombólise, ATPC ou RM. Todos os exames foram realizados em nosso centro de referência de cardiologia durante a fase aguda do evento coronariano, o Instituto do Coração (InCor), localizado aproximadamente oito quilômetros do HU-USP. O HU-USP não é um hospital especializado e, por isso, nem procedimentos de cateterismo cardíaco nem RM era disponível. ^10^

### Definição de síndrome coronariana aguda

Todos os pacientes com suspeita de SCA no departamento de emergência do HU-USP foram rastreados para participar no ERICO. Os critérios de elegibilidade para inclusão no estudo foram: diagnóstico de infarto do miocárdio com elevação do segmento ST (IAMSST), infarto agudo do miocárdio sem elevação do segmento ST (IAMSSST), ou de angina instável (AI). Os critérios usados para definir SCA foram: ^9 - 12^

1) Infarto do miocárdio: presença de sintomas consistentes com isquemia cardíaca dentro de 24 horas da admissão e níveis de troponina I acima do percentil 99, com um coeficiente de variação específico < 10%.
1a) IAMSST: presença de critérios para infarto do miocárdio, mais um dos seguintes critérios: elevação persistente do segmento ST igual ou superior a 1mm em duas derivações eletrocardiográficas contíguas, ou presença de um bloqueio novo ou presumivelmente novo do ramo esquerdo.
1b) IAMSSST: presença de critérios para infarto do miocárdio, mas não para IAMSST.
2) AI: sintomas consistentes com isquemia cardíaca 24 horas antes da admissão hospitalar, ausência de critérios para infarto do miocárdio e ao menos um dos seguintes critérios: história de DAC; teste de estratificação de risco cardíaco positivo (invasivo ou não invasivo); alterações transitórias do segmento ST igual ou superior a 0,5mm em duas derivações contíguas, nova inversão da onda T igual ou superior a 1 mm e/ou pseudonormalização de ondas T previamente invertidas; troponina I igual ou superior a 0,4ng/mL (que garante níveis de troponina I acima do percentil 99 independentemente do kit utilizado); ou concordância diagnóstica de dois médicos independentes.

### Protocolo do estudo

Na admissão hospitalar por SCA, após assinar o termo de consentimento, todos os participantes forneceram informações basais de acordo com questionários padronizados que incluíram dados sociodemográficos, fatores de risco cardiovasculares (hipertensão, diabetes, obesidade, dislipidemia, tabagismo, história pessoal ou familiar de DAC, sedentarismo, uso de cocaína e menopausa), e uso prévio de medicamentos. As condições clínicas foram relatadas pelos próprios pacientes.

Três pacientes foram responsáveis, de maneira independente, pela revisão das informações e validação dos casos de SCA. O protocolo do estudo também incluiu amostras de sangue para exames laboratoriais, incluindo troponina I, creatina quinase MB, hemograma e lipidograma (colesterol total, HDL-colesterol, LDL-colesterol e triglicerídeos).

Após 30 dias do evento agudo, todos os participantes foram convidados para atualizar suas informações sobre os fatores de risco cardiovasculares. Aos seis meses, e anualmente após o evento inicial, os pacientes foram contactados por telefone para atualizar suas informações, ocorrência de óbito, história cardiovascular, e uso de medicamentos. Sempre que o paciente relatasse um novo evento de SCA potencial, iniciava-se uma investigação para se obter mais informações. Outros detalhes do ERICO foram descritos anteriormente. ^10^

### Desfechos

Informações sobre mortalidade por todas as causas, mortalidade por DCV e mortalidade por DAC foram obtidas do ERICO. A ocorrência de óbito foi atualizada pelos prontuários médicos e certificados de óbito. Os dados de mortalidade foram confirmados por certificados de óbito obtidos do sistema de estatística de São Paulo (PRO-AIM - Programa de Aprimoramento das Informações de Mortalidade no município de São Paulo), agências de saúde do estado de São Paulo (fundação SEADE - Sistema Estadual de Análise de Dados) e o Ministério da Saúde. Regularmente, a equipe da pesquisa preparou uma lista de indivíduos que eram descritos como mortos ou cujos contatos haviam sido perdido. As agências de saúde estadual e municipal buscaram pelos certificados de óbito em seus bancos de dados e relataram os resultados à equipe do ERICO. Em nosso estudo, usamos a causa básica da morte e, se necessário, reclassificamos a causa subjacente de morte. Qualquer discordância entre eles era resolvida por discussão com um terceiro revisor. Causa cardiovascular de morte foi definida como qualquer diagnóstico classificado em “doenças do sistema circulatório” da Classificação Internacional de Doenças versão 10 (CID-10), capítulo IX, ou como CID-10 código R57.0 “choque cardiogênico”. Cada evento foi classificado usando critérios internacionais pré-definidos. ^13 , 14^ A mortalidade dos participantes foi classificada como “mortalidade pós-infarto do miocárdio” sempre que DAC fosse identificada como a principal causa de morte. Para tanto, usou-se a definição de infarto do miocárdio (CID-10 código I21.X). Mortalidade por todas as causas referiu-se à morte independente da causa subjacente registrada.

O protocolo do estudo foi aprovado pelo comitê de ética institucional para pesquisa envolvendo seres humanos. Todos os participantes assinaram um termo de consentimento de inclusão no estudo.

### Análise estatística

Estatística descritiva dos participantes é apresentada por estratégia terapêutica (tratamento clínico exclusivo como grupo de referência, ATPC e RM). As variáveis categóricas, apresentadas como frequências relativas e absolutas, foram analisadas usando o teste do qui-quadrado. Uma vez que não foi observada nenhuma distribuição paramétrica pelo teste de normalidade Kolmogorov-Smirnov, as variáveis contínuas são apresentadas como medianas e respectivos intervalos interquartis (IIQs) e comparadas entre os grupos de tratamento pelo teste de Kruskal-Wallis.

Análises de sobrevida foram realizadas por curvas de Kaplan-Meier ^15^ e modelo de riscos proporcionais de Cox [ *hazard ratio* (HR) com respectivos intervalos de confiança (IC)] ^16^ para avaliar mortalidade por todas as causas, por DCV e por DAC de acordo com as estratégias terapêuticas (tratamento medicamentoso exclusivo como grupo de referência, ATPC e RM). Os pacientes foram acompanhados por sete anos, com um período mediano de acompanhamento de 1460 dias (quatro anos). Assim, a análise de regressão de Cox e de *hazard ratio* foram conduzidas em 180 dias e anualmente até quatro anos após um evento agudo. Os seguintes modelos de regressão de Cox foram calculados: sem ajuste, ajustado por idade, e ajustado quanto história de DAC, subtipo da DAC (AI, IAMSST, IAMSSST), tabagismo (passado, atual ou ausente), hipertensão (sim/não), diabetes (sim/não), dislipidemia (sim/não), fração de ejeção, e de acordo com o número de artérias coronárias maiores obstruídas (≥ 50%), ou de qualquer de seus ramos [artéria descendente anterior, artéria circunflexa e artéria coronária direita] – sem obstrução (todos os vasos com obstrução < 50%), um vaso (obstrução ≥ 50% em uma artéria coronária principal ou seus ramos principais), dois vasos (obstrução ≥ 50% em duas artérias coronárias principais ou seus ramos principais) e doença de múltiplos vasos (obstrução ≥50% em duas artérias coronárias principais ou seus ramos principais) ou na artéria coronária principal esquerda, ou presença de RM prévia.

Também restringimos nossas análises para os casos de IAMSSST para esclarecer a associação entre mortalidade em longo prazo e intervenção terapêutica. Todos os testes foram bicaudais, como significância <0,05. As análises foram conduzidas usando o programa SPSS® Statistics versão 25,0 do IBM®.

## Resultados

### Características clínicas e sociodemográficas

Características basais dos participantes do ERICO por estratégia terapêutica após SCA são apresentadas na Tabela 1 . A idade mediana dos pacientes foi 62 ± 12,9 anos, a maioria dos participantes eram homens (n=493; 61,6%), brancos (n= 536; 67,2%), casados (n=506; 63,5%) e baixa escolaridade (62,8% tinham ensino fundamental). O tempo mediano entre o início dos sintomas de SCA e a primeira intervenção (ATPC ou RM) foi de quatro dias (IIQ: 1-8 dias) durante a internação no HU-USP (mediana oito dias; IIQ: 4-13 dias), sem diferença significativa entre os submetidos à ATPC e à RM. Em relação às estratégias terapêuticas, 343 (42,9%) pacientes se submeteram ao tratamento clínico exclusivo (15 haviam se submetido à trombólise anteriormente), 400 (50,0%) pacientes submeteram-se à ATPC ou colocação de cateter (65 haviam se submetido à trombólise anteriormente), e 57 (7,1%) submeteram-se à RM (um havia se submetido à trombólise previamente). Observamos que os participantes submetidos à eram mais velhos e apresentavam níveis um pouco mais altos de colesterol total, colesterol LDL e triglicerídeos. No entanto, frequências mais altas de tabagismo atual e IAMSST foram encontradas entre os indivíduos submetidos à ATPC em comparação aos outros grupos. Embora uma frequência mais alta de pacientes que relataram história de insuficiência cardíaca antes do evento índice foi encontrada entre aqueles submetidos à terapia clínica isolada, a fração de ejeção não foi estatisticamente diferente entre os grupos de tratamento ( Tabela 1 ).

**Tabela 1:** – Características basais dos 800 participantes do ERICO de acordo com as estratégias terapêuticas

Características sociodemográficas	Tratamento medicamentoso (n=343)	ATPC (n=400)	RM (n=57)	Total (n=800)	Valor p
Idade mediana, anos (IIQ)	62 (54-71)	61 (52-70)	65 (58-74)	61 (53-71)	0,04
Homens (%)	198 (57,7)	259 (64,8)	36 (63,2)	493 (61,6)	0,14
**Raça (%)**					0,63
Branca	222 (64,7)	273 (68,6)	41 (71,9)	536 (67,2)	
Parda	99 (28,9)	103 (25,9)	11 (19,3)	213 (26,7)	
Negra	20 (5,8)	18 (4,5)	4 (7)	42 (5,3)	
Amarela	2 (0,6)	4 (1)	1 (1,8)	7 (0,9)	
**Estado civil (%)**					0,20
Solteiro	40 (11,8)	53 (13,3)	7 (12,3)	100 (12,5)	
Casado	204 (60)	267 (66,8)	35 (61,4)	506 (63,5)	
Divorciado	32 (9,4)	32 (8)	7 (12,3)	71 (8,9)	
Viúvo(a)	64 (18,8)	48 (12)	8 (14)	120 (15,1)	
**Educação (%)**					0,65
Sem educação formal	47 (13,7)	43 (10,8)	6 (10,5)	96 (12)	
Ensino fundamental	214 (62,4)	248 (62)	40 (70,2)	502 (62,8)	
Ensino médio	57 (16,6)	71 (17,8)	8 (14)	136 (17)	
Ensino superior	25 (7,3)	38 (9,5)	3 (5,3)	66 (8,3)	
**Fatores de risco cardiovasculares**					
Tabagismo (%)					<0,001
Atual	78 (23,8)	141 (36,3)	9 (17,0)	228 (29,6)	
Passado	133 (40,5)	148 (38,1)	25 (47,2)	306 (39,8)	
Nunca	117 (35,7)	99 (25,5)	19 (35,8)	235 (30,6)	
IMC, Kg/m ^2^ mediana (IIQ)	27 (24-30)	27 (24-30)	26 (25-30)	27 (24-30)	0,55
Obesidade (%)	79 (24,3)	99 (25,9)	11 (23,9)	189 (25,1)	0,87
Hipertensão (%)	256 (75,5)	286 (72,8)	46 (80,7)	588 (74,5)	0,38
Diabetes (%)	139 (41,1)	140 (36,2)	28 (50)	307 (39,3)	0,09
Dislipidemia (%)	155 (51)	197 (55,2)	33 (64,7)	385 (54,1)	0,16
Sedentarismo (%)	222 (69,2)	256 (67,4)	43 (78,2)	521 (68,9)	0,27
^*^ Colesterol total, mg/dL	172 (141-208)	162 (134-204)	180 (148-212)	177 (148-210)	0,02
^*^ LDL Colesterol, mg/dL	103 (78-132)	96 (75-131)	108 (81-134)	108 (80-126)	0,04
HDL colesterol, mg/dL	37 (31-44)	37 (31-44)	37 (31-43)	38 (32-50)	0,51
Triglicerídeos, mg/dL	133 (98-189)	123 (94-176)	147 (106-202)	123 (90-162)	0,04
**Tipos de SCA (%)**					<0,0001
Angina	108 (31,5)	69 (17,3)	17 (29,8)	194 (24,3)	
IAMSSST	160 (46,6)	159 (39,8)	32 (56,1)	351 (43,9)	
AMSST	75 (21,9)	172 (43)	8 (14)	255 (31,9)	
DAC prévia (%)	88 (27,8)	80 (21,2)	14 (25,5)	182 (24,3)	0,13
FEVE	58 (43-67)	58 (45-67)	60 (44-65)	58 (45-67)	0,34
^*^ Insuficiência cardíaca (%)	69 (24,0)	55 (15,5)	2 (4,5)	126 (18,3)	0,001

* Todas essas comorbidades foram autorrelatadas por participantes com base no diagnóstico clinico e tratamento. ATPC: angioplastia transluminal percutânea coronaria; RM: revascularização do miocárdio; IMC: índice de massa corporal; DAC: doença arterial coronariana; IAMSSST: infarto agudo do miocárdio sem supradesnivelamento do segmento ST; IAMSST: infarto agudo do miocárdio com supradesnivelamento do segmento ST; FEVE: fração de ejeção do ventrículo esquerdo. Todas as variáveis contínuas foram descritas como mediana e intervalo interquartil (IIQ). Os valores p foram obtidos do teste do qui-quadrado ou o teste Kruskal-Wallis para variáveis categóricas, respectivamente

Em relação à terapia medicamentosa na admissão, os pacientes submetidos a RM apresentaram a porcentagem mais baixa de uso de varfarina (3,6%; p=0,032) em comparação aos demais grupos. A maior frequência de uso de clopidogrel em 30 dias foi observada entre os pacientes submetidos à ATPC (51,5%; p= 0,018), e os pacientes submetidos a RM apresentaram maior uso de bloqueador do canal de cálcio (20%; p=0,029). Em 180 dias, os pacientes submetidos à ATPC apresentaram a maior frequência de uso de clopidogrel em comparação aos outros grupos (30%, p=0,014). Em um ano, não observamos diferenças estatisticamente quanto ao uso de medicamentos (Tabela Suplementar 1).

### Terapia de reperfusão versus obstrução coronária

Em relação à extensão da doença obstrutiva, encontramos 107 (13,4%) pacientes sem obstrução, 304 (38,0%) pacientes com doença em um único vaso, 169 (21,1%) com doença de dois vasos e 220 (27,5%) com doença em múltiplos vasos. Apesar de a maioria dos pacientes incluídos no grupo “terapia medicamentosa exclusiva” ter apresentado doença coronária leve, eles apresentaram uma maior frequência de doença de múltiplos vasos em comparação ao grupo submetido à ATPC (28,9% vs. 18,0%, p<0,0001).

A maioria dos 400 pacientes submetidos à ATPC recebeu *stents* de metal (n=325; 75,8%) seguido de angioplastia com balão (n=57; 13,3%) e *stent* farmacológico (n=47; 10,9%). Vale mencionar que, entre os pacientes submetidos à ATPC, somente 40 pacientes (10%) necessitaram de nova revascularização por ATPC, e três pacientes (0,75%) necessitaram de RM após a ATPC. Nenhum paciente submetido a RM necessitou de uma nova revascularização.

### Taxas de mortalidade e de sobrevida

**Figura 1:**
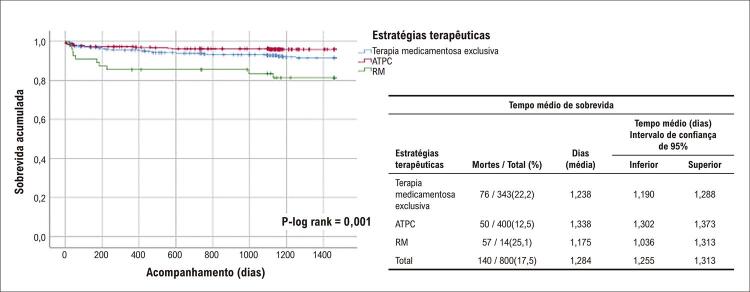
– Curva de sobrevida de Kaplan-Meier para mortalidade por todas as causas durante o acompanhamento por quatro anos. ATPC: angioplastia transluminal percutânea coronaria; RM: revascularização do miocárdio.

**Figura 2 f03:**
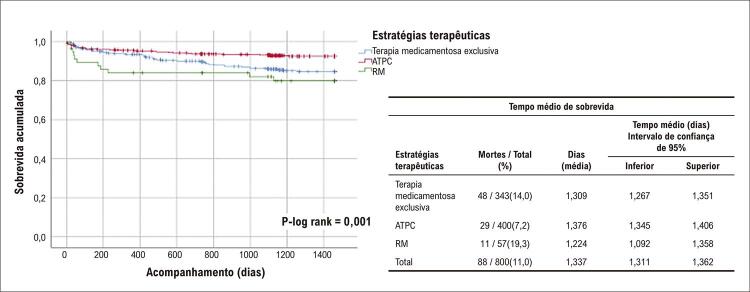
– Curva de sobrevida de Kaplan-Meier para mortalidade por doença cardiovascular durante o acompanhamento por quatro anos. ATPC: angioplastia transluminal percutânea coronaria; RM: revascularização do miocárdio.

**Figura 3 f04:**
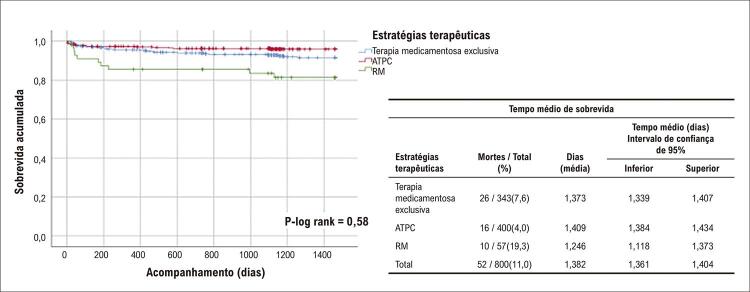
– Curva de sobrevida de Kaplan-Meier para mortalidade por infarto do miocárdio durante o acompanhamento por quatro anos. ATPC: angioplastia transluminal percutânea coronaria; RM: revascularização do miocárdio.

No geral, observamos 140 mortes após a SCA (88 óbitos por DCV, 52 por DAC). Nós observamos as seguintes taxas de letalidade: terapia medicamentosa exclusiva (76/274; 27,7%), ATPC (50/314; 15,9%) e 14/48 (29,2%), p=0,001 até quatro anos de seguimento. As piores taxas de sobrevida também foram observadas entre os indivíduos submetidos a RM: mortalidade por todas as causas e DCV, p-log rank 0,001, Figuras 1-3 [Fig f03] [Fig f04] .

Nos modelos de regressão de Cox, a ATPC foi associada a menor probabilidade de eventos fatais após ajuste multivariado durante os quatro anos de acompanhamento. Todas as causas [HR: 0,42 (IC95% 0,26-0,70], DCV [HR: 0,39 (IC95% 0,20-0,73)] e DAC [HR multivariado 0,24 (IC95% 0,09-0,63)] em comparação aos submetidos à tratamento medicamentoso exclusivo. O procedimento de RM foi correlacionado à mortalidade por DAC após ajuste por idade e sexo [HR: 2,19 (IC95% 1,05-4,55)]. No entanto, o risco perdeu significância no modelo multivariado ( Tabela 2 ).

**Tabela 2:** – Hazard ratios (HR) [Intervalos de confiança de 95% (IC95%)] de mortalidade por todas as causas, doenças cardiovasculares e doença arterial coronariana dos 800 participantes do ERICO segundo estratégias de tratamento após um evento coronariano agudo

Mortalidade por todas as causas (Total de mortes =140)	180 dias	1 ano	2 anos	3 anos	4 anos
**Sem ajuste**					
Tratamento medicamentoso	Referência	Referência	Referência	Referência	Referência
ATPC	0,74 (0,41 - 1,35)	0,62 (0,36 - 1,04)	0,53 (0,35 - 0,82)	0,51 (0,34 - 0,75)	0,53 (0,37 - 0,75)
RM	2,21 (0,99 - 4,93)	2,12 (1,07 - 4,20)	1,32 (0,69 - 2,53)	1,24 (0,68 - 2,25)	1,15 (0,65 - 2,03)
**Ajustado por idade e sexo**					
Tratamento medicamentoso	Referência	Referência	Referência	Referência	Referência
ATPC	0,79 (0,43 - 1,43)	0,64 (0,38 - 1,09)	0,55 (0,36 - 0,85)	0,52 (0,35 - 0,77)	0,55 (0,38 - 0,78)
RM	2,02 (0,90 - 4,51)	1,91 (0,96 - 3,78)	1,18 (0,62 - 2,26)	1,11 (0,61 - 2,01)	1,04 (0,59 - 1,84)
**Ajuste multivariado ^**1**^**					
Tratamento medicamentoso	Referência	Referência	Referência	Referência	Referência
ATPC	0,52 (0,22 - 1,25)	0,46 (0,21 - 0,99)	0,38 (0,21 - 0,69)	0,41 (0,24 - 0,7)	0,42 (0,26 - 0,70)
RM	0,90 (0,25 - 3,21)	1,10 (0,4 - 2,99)	0,59 (0,23 - 1,54)	0,64 (0,27 - 1,54)	0,62 (0,28 - 1,39)
**Mortalidade por DCV (Total de mortes = 88)**					
**Sem ajuste**					
Tratamento medicamentoso	Referência	Referência	Referência	Referência	Referência
ATPC	0,81 (0,41 - 1,59)	0,69 (0,37 - 1,3)	0,61 (0,37 - 1,03)	0,50 (0,31 - 0,81)	0,49 (0,31 - 0,78)
RM	2,61 (1,08 - 6,29)	2,60 (1,2 - 5,65)	1,68 (0,81 - 3,51)	1,43 (0,72 - 2,84)	1,44 (0,75 - 2,77)
**Ajustado por idade e sexo**					
Tratamento medicamentoso	Referência	Referência	Referência	Referência	Referência
ATPC	0,84 (0,43 - 1,67)	0,72 (0,38 - 1,34)	0,63 (0,38 - 1,06)	0,51 (0,32 - 0,83)	0,50 (0,31 - 0,79)
RM	2,36 (0,98 - 5,69)	2,31 (1,06 - 5,03)	1,49 (0,71 - 3,11)	1,25 (0,63 - 2,49)	1,27 (0,66 - 2,45)
**Ajuste multivariado ^**1**^**					
Tratamento medicamentoso	Referência	Referência	Referência	Referência	Referência
ATPC	0,56 (0,2 - 1,54)	0,54 (0,22 - 1,32)	0,46 (0,22 - 0,94)	0,40 (0,20 - 0,79)	0,39 (0,20 - 0,73)
RM	1,15 (0,31 - 4,26)	1,17 (0,37 - 3,66)	0,77 (0,26 - 2,27)	0,83 (0,31 - 2,22)	0,84 (0,34 - 2,07)
**Mortalidade por DAC (Total de mortes = 52)**					
**Sem ajuste**					
Tratamento medicamentoso	Referência	Referência	Referência	Referência	Referência
ATPC	0,86 (0,37 - 1,98)	0,62 (0,29 - 1,36)	0,63 (0,32 - 1,23)	0,57 (0,29 - 1,10)	0,51 (0,27 - 0,94)
RM	3,44 (1,27 - 9,29)	3,38 (1,43 - 7,96)	2,54 (1,12 - 5,76)	2,58 (1,19 - 5,61)	2,42 (1,16 - 5,01)
**Sem ajuste por idade e sexo**					
Tratamento medicamentoso	Referência	Referência	Referência	Referência	Referência
ATPC	0,91 (0,39 - 2,10)	0,65 (0,30 - 1,41)	0,65 (0,33 - 1,28)	0,59 (0,30 - 1,13)	0,52 (0,28 - 0,97)
RM	3,16 (1,17 - 8,57)	3,06 (1,3 - 7,25)	2,29 (1,00 - 5,2)	2,31 (1,06 - 5,03)	2,19 (1,05 - 4,55)
**Ajuste multivariado ^**1**^**					
Tratamento medicamentoso	Referência	Referência	Referência	Referência	Referência
ATPC	0,49 (0,12 - 2,02)	0,39 (0,10 - 1,50)	0,31 (0,10 - 0,91)	0,29 (0,10 - 0,84)	0,24 (0,09 - 0,63)
RM	0,85 (0,17 - 4,21)	1,01 (0,26 - 3,93)	0,76 (0,21 - 2,75)	0,91 (0,29 - 2,86)	0,93 (0,33 - 2,58)

*^*1*^ Ajustada por idade, sexo, tipo de síndrome coronariana aguda (angina instável, infarto agudo do miocárdio sem supradesnivelamento do segmento ST, infarto agudo do miocárdio com supradesnivelamento do segmento, história de doença arterial coronariana prévia, tabagismo (atual, passado ou nunca), hipertensão (sim/não), diabetes (sim/não), e fração de ejeção de acordo com o número de artérias principais ou quais de seus ramos obstruídos (≥ 50%) (sem obstrução, um vaso, dois vasos, múltiplos vasos); ATPC: angioplastia transluminal percutânea coronaria; RM: revascularização do miocárdio.*

Nós selecionamos os pacientes que se submeteram à terapia medicamentosa exclusiva como o grupo de referência por haver menos indivíduos com as formas mais graves de SCA (angina n=108; 31,5%), e o número mais alto de pacientes sem obstrução (n=103; 96,3%).

Ajustes adicionais para o uso de medicamentos como aspirina, agentes hipolipemiantes, inibidores de enzima conversora de angiotensina, betabloqueadores, ou varfarina no basal e após o evento agudo não alteraram nossos achados. Para ATPC, os HRs da mortalidade em quatro anos foram: todas as causas 0,39 (IC95% 0,25-0,61), DCV 0,36 (95% CI: 0,20-0,65) e DAC 0,31 (IC95% 0,14-0,70). Para RM, os HRs da mortalidade foram: todas as causas 0,91 (IC95% 0,47-1,76), DCV 1,06 (IC95% 0,49-2,32) e DAC 1,27 (IC95% 0,53-3,05).

Ao restringir nossas análises aos 351 casos de IAMSSST (80 óbitos), encontramos resultados similares quanto à associação inversa com riscos de mortalidade a longo prazo e ATPC em comparação aos pacientes submetidos à terapia medicamentosa exclusiva (Tabela Suplementar 2). De fato, os HRs para DCV e DAC em nosso estudo foram ainda mais baixos para pacientes com IAMSSST submetidos à ATPC [risco em quatro anos: DCV 0,28 (IC95% 0,12-0,67) e DAC 0,13 (IC95% 0,03-0,63) em comparação aos mesmos riscos de mortalidade observados quando incluímos toda a amostra no modelo de Cox.

## Discussão

### Principais resultados sobre evento coronário agudo e mortalidade

Em nossa amostra, o grupo submetido à ATPC foi o único que mostrou associação significativa com mortalidade por todas as causas, por DCV e por DAC mais baixas durante os quatro anos de acompanhamento ( Figura central ). Alguns estudos que avaliaram intervenções terapêuticas após um evento de SCA restringiram seus estudos a pacientes com IAMSSST e não encontraram associação significativa ao comparar estratégias invasivas com estratégias mais conservadoras. ^5 - 7^

**Figura Central:**
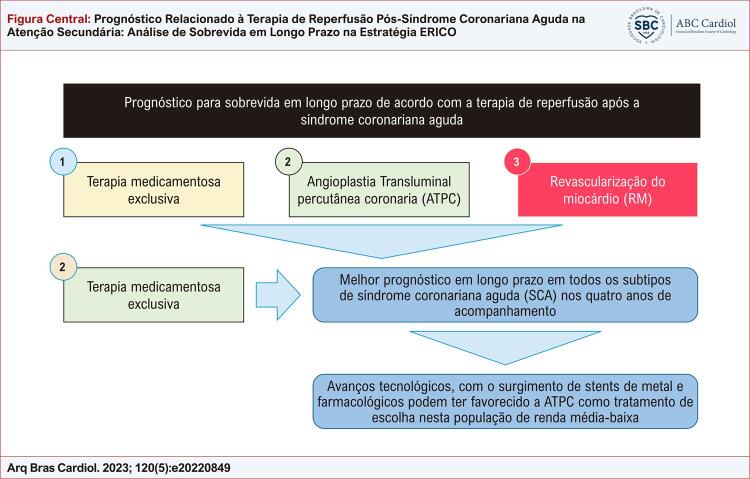
: Prognóstico Relacionado à Terapia de Reperfusão Pós-Síndrome Coronariana Aguda na Atenção Secundária: Análise de Sobrevida em Longo Prazo na Estratégia ERICO

Em um ensaio clínico randomizado (ECR) de Boden et al., ^5^ 920 pacientes com IAMSSST foram separados aleatoriamente em dois grupos (tratamento invasivo e tratamento conservador), sem diferenças significativas quanto à mortalidade por todas as causas durante os 23 meses de seguimento. Em outro ECR, McCullough et al. ^6^ estudaram 201 pacientes com suspeita de SCA, que eram inelegíveis para trombólise, e não encontraram diferenças significativas entre os grupos de tratamento durante 21 meses de acompanhamento. ^6^ Outro ECR com 313 pacientes com IAMSSST alocados em dois grupos – estratégia precoce agressiva ou estratégia inicialmente conservadora – também não encontrou diferenças significativas quanto à mortalidade durante acompanhamento por 12 meses. ^7^

Em nosso estudo, quando restringimos nossa amostra a pacientes com IAMSSST, aqueles submetidos à ATPC apresentaram mortalidade por toda as causas, por DCV e por DAC mais baixa em comparação aos submetidos à terapia medicamentosa exclusiva. Vale mencionar que mais de 50% das mortes na amostra total ocorreram entre os pacientes com IAMSSST (80/140 das mortes totais); assim, esse subgrupo foi o que mais se beneficiou da ATPC. E diferentemente dos estudos anteriores, ^5 , 6^ consideramos um período mais longo de acompanhamento que nos permitiu detectar um efeito benéfico da ATPC na mortalidade por todas as causas, por DCV e por DAC em todos os subtipos de SCA.

Os pacientes do ERICO foram inicialmente atendidos em um hospital terciário e, assim, o intervalo entre o início dos sintomas e a intervenção cardíaca na maioria dos casos foi mais longo (após 24 – 48 horas) em comparação a de estudos conduzidos em centros especializados, em que os procedimentos cardiológicos são geralmente realizados dentro de 24 a 48 horas após um evento agudo. ^5 - 7^ Mesmo considerando essa limitação, nosso principal resultado apoia a ATPC para os casos de IAMSSST. Ainda, a trombólise química previamente realizada na fase aguda em 16,3% do subgrupo da ATPC também possa ter contribuído para um melhor prognóstico em longo prazo em nosso estudo, mesmo com as frequências mais altas de IAMSST e tabagismo atual nesse subgrupo, em comparação a outros subgrupos (RM e terapia medicamentosa exclusiva).

Outra possível explicação para o efeito protetor em longo prazo observado no subgrupo da ATPC é a frequência mais alta de *stents* metálicos (75,8% dos casos de ATPC). De fato, com o advento do stent de metal, a necessidade de reestenose tornou-se menos frequente. ^17 , 18^ Estudos recentes mostraram uma taxa mais baixa de revascularização em pacientes que receberam *stents* de metal (19,8%) e *stents* farmacológicos (16,5%) [HR: 0,76 (IC95% 0,69-0,85) p<0,001], mas sem diferenças significativas na mortalidade por todas as causas ou infarto do miocárdio não fatal durante os seis meses de acompanhamento. Em nosso estudo, a taxa de revascularização após a primeira ATPC foi ainda mais baixa (10%).

Ainda, nossos achados diferem-se dos da literatura, o que pode ser explicado pelo fato de nossa amostra haver incluído todos os tipos de SCA, e não somente pacientes de alto risco com doença de múltiplos vasos. Porém, mesmo considerando os casos de IAMSSST, nossos resultados foram confirmados. Ainda, nossos resultados refletem um período que testemunhou três eras de *stents* (angioplastia com balão, *stent* de metal, e *stent* farmacológico), o que pode explicar por que nossos resultados corroboram a ATPC como uma terapia invasiva precoce, relacionada com melhor sobrevida em longo prazo nesta população de baixa renda.

Há apenas um estudo mostrando associações significativas entre SCA e mortalidade. Wijeysundera et al. ^8^ avaliaram 50302 pacientes com IAMSSST segundo o tratamento de reperfusão sete dias após a angiografia de referência: 68,2% foram submetidos à revascularização [ATPC (n=28011) e RM (n=6,227)] ou terapia medicamentosa exclusiva (n=16014). Durante o acompanhamento de seis anos, os pacientes submetidos a RM [HR: 0,53 (IC95% 0,47–0,60)] e ATPC [HR: 0,64 (IC95% 0,60–0,69)] apresentaram menor risco de mortalidade em comparação ao subgrupo submetidos à terapia medicamentosa exclusiva.

Em nosso estudo, os pacientes que se submeteram a RM (n=52) após o evento índice apresentaram as taxas mais baixas de sobrevida para mortalidade em quatro anos de acompanhamento em modelos sem ajustes e modelos ajustados por idade e sexo. Contudo, esses achados não foram confirmados nos modelos multivariados. Provavelmente, o pequeno número de casos de RM não nos permitiu detectar significância estatística em nossa análise multivariada para mortalidade por todas as causas, DCV e DAC, como observamos para o subgrupo ATPC.

### Pontos fortes do estudo

Este estudo oferece uma oportunidade única de avaliar a sobrevida e a mortalidade (todas as causas, DCV e DAC) em longo prazo, de acordo com as estratégias de tratamento adotadas após a SCA em uma população de renda média-baixa atendida no serviço secundário. Ainda, a maioria dos estudos anteriores não descreveram os efeitos do tratamento clínico sobre a mortalidade específica, como nós fizemos.

### Limitações

Por se tratar de um estudo observacional, não podemos extrapolar nossos achados a outras populações. Além disso, há fatores de confusão que não podem ser controlados, incluindo viés de seleção das estratégias de tratamento. Além disso, a angiografia invasiva para o diagnóstico de obstrução coronária não foi realizada por um único profissional ou por uma equipe restrita de profissionais. No entanto, um cardiologista do estudo ERICO revisou todos os casos cuidadosamente e fez a classificação de acordo com a extensão da doença obstrutiva.

Finalmente, embora tenhamos observado uma taxa baixa de nova intervenção cardiológica nos subgrupos (menos que 1% dos participantes necessitaram de RM após ATPC) nos quatro anos de acompanhamento, a possibilidade de que taxas mais altas possam ser encontradas em um acompanhamento mais longo não pode ser excluída.

## Conclusões

No estudo ERICO, a estratégia de reperfusão por ATPC foi associada com melhor prognóstico em longo prazo em todos os subtipos de SCA durante os quatro anos de acompanhamento. Nossos achados foram confirmados mesmo considerando somente os casos de IAMSSST. O avanço da tecnologia com a emergência de *stents* de metal e farmacológicos pode ter favorecido a ATPC como tratamento de escolha nesta população de renda média-baixa.

## * Material suplementar

Para informação adicional, por favor, clique aqui .
