# Supplementary material for: Prognóstico Relacionado à Terapia de Reperfusão Pós-Síndrome Coronariana Aguda na Atenção Secundária: Análise de Sobrevida em Longo Prazo na Estratégia ERICO
Source: Arq Bras Cardiol. 2023 May 2;120(5):e20220849. [Article in Portuguese] doi: 10.36660/abc.20220849 (PMC10263393; doi:10.36660/abc.20220849)
Supplement: Translation

# Translation: Prognosis Related to Reperfusion Therapy Post-Acute Coronary Syndrome in Secondary Care: Long-Term Survival Analysis in the ERICO Study

**Authors:** Tatiana C. Bruno, Márcio S. Bittencourt, Alessandra V. L. Quidim, Itamar S. Santos, Paulo A. Lotufo, Isabela M. Benseñor, Alessandra C. Goulart

**Affiliations:** 1 Brazil Centro de Pesquisa Clínica e Epidemiológica – Hospital Universitário – Universidade de São Paulo, São Paulo, SP – Brazil; 2 Brazil Universidade de São Paulo, Faculdade de Medicina, São Paulo, SP – Brazil

**Keywords:** Acute Coronary Syndrome, Mortality, Survival Analysis, Angioplasty, Coronary Artery Bypass

## Abstract

**Background:**

Relationship between reperfusion therapy post-acute coronary syndrome (ACS) and mortality in secondary care is not well-known.

**Objectives:**

To evaluate the impact of three therapeutic strategies: (1) exclusive medical therapy, (2) percutaneous coronary intervention (PCI) and (3) coronary artery bypass grafting (CABG) on long-term survival of participants in the Strategy of Registry of Acute Coronary Syndrome (ERICO) study.

**Methods:**

Survival analyses for all-cause, cardiovascular (CVD) and coronary artery disease (CAD) mortality were performed according to three therapeutic strategies (exclusive medical therapy, PCI or CABG). Cox regression models were used to estimate the hazard ratio (HR) with respective 95% confidence interval (95%CI) from 180 days to four years of follow-up after ACS. Models are presented as crude, age-sex adjusted and further adjusted for previous CAD, ACS subtype, smoking, hypertension, dyslipidemia, left ventricular ejection fraction and according to the number of obstructed (≥ 50%) major coronary arteries.

**Results:**

Among 800 participants, the lowest crude survival rates were detected among individuals who underwent CABG (all-cause and CVD). CABG was correlated to CAD (HR: 2.19 [95% CI: 1.05-4.55]). However, this risk lost significance in the full model. PCI was associated to lower probability of fatal events during four-year follow-up: all-cause [multivariate HR: 0.42 (95% CI: 0.26-0.70)], CVD [HR: 0.39 (95% CI: 0.20-0.73)] and CAD [multivariate HR: 0.24 (95% CI: 0.09-0.63)] compared to those submitted to exclusive medical therapy.

**Conclusion:**

In the ERICO study, PCI after ACS was associated to better prognosis, particularly CAD survival.

## Introduction

Cardiovascular disease (CVD) is responsible for high mortality rates, particularly in low and middle income countries (LMICs).^1 , 2^In Brazil, CVD was the main cause of death in the last decades, with coronary artery disease (CAD) accounting for one third of deaths for CVD in 2016.^3^

Despite advances in cardiovascular treatment with surgical techniques and the emerging of drug-eluting stents,^4^recent studies that compared different therapeutic strategies after an acute coronary syndrome (ACS), such as percutaneous coronary intervention (PCI), coronary artery bypass grafting (CABG), and exclusive medical therapy have reported controversial findings regarding their impact on mortality.^5 - 8^In LMICs, socioeconomic inequalities coupled with a lack of access to tertiary cardiology centers represent a barrier to more effective treatment during the acute phase of a coronary event. This lack of adequate treatment have contributed to the high mortality rates in Brazil in the last decades.^9^Thus, we evaluated the long-term prognosis of ACS patients, participants of the Strategy of Registry of Acute Coronary Syndrome (ERICO) study according to the therapy implemented after ACS. We compared three therapeutic strategies: (1) exclusive medical therapy, (2) PCI (balloon angioplasty, bare metal, and drug-eluting stents) and (3) CABG.

## Methods

### Sample design and population

All patients were participants in the ERICO study, a prospective cohort study of ACS individuals recruited at the University Hospital of the University of São Paulo (HU-USP) from February 2009 to December 2013. Further details of the ERICO study are described elsewhere.^10^

In brief, ERICO is an ongoing study at the HU-USP, a 260-bed secondary hospital located in Sao Paulo at the district of Butantan, which had a population of 428,000 inhabitants in 2010.^9 , 11^Although Butantan has some socioeconomic indicators above the city’s average (e.g., average family income), it is characterized by wide inequalities.^11^Of note, ERICO participants live in a low-middle income neighborhood and may have difficulties to access health care services.

Here, we evaluated all participants (n=800/1085, 73.7%), admitted at the emergency department of HU-USP, with confirmed ACS submitted to invasive angiography for the diagnosis of coronary obstruction, followed by therapy after the acute phase with exclusive medical therapy, including thrombolysis, PCI or CABG. All exams were performed in our main cardiology referral center during acute phase of coronary event, the Instituto do Coração (InCor), located approximately eight kilometers away from HU-USP. Since HU-USP is a non-specialized hospital, neither cardiac catheterization procedures nor CABG was available.^10^

### Definition of acute coronary syndrome

All patients with suspected ACS at the emergency department of HU-USP were screened to participate in the ERICO study. Eligibility inclusion criteria included a diagnosis of ST elevation myocardial infarction (STEMI), non-ST elevation myocardial infarction (NSTEMI) or unstable angina (UA). The criteria used to define ACS were:^9 - 12^

1) Myocardial infarction: presence of symptoms consistent with cardiac ischemia within 24 hours of hospital presentation and troponin I levels above the 99^th^percentile with a test-specific coefficient of variation < 10%.
1a) STEM: presence of criteria for myocardial infarction plus one of the following: persistent ST segment elevation equal to or greater than 1mm in two contiguous electrocardiographic leads or the presence of a new or presumably new left bundle branch block.
1b) NSTEMI: presence of criteria for myocardial infarction but not for STEMI.
2) UA: symptoms consistent with cardiac ischemia 24 hours prior to hospital admission, absence of myocardial infarction criteria and at least one of the following: history of CAD; positive cardiac risk stratification test (invasive or noninvasive); transient ST segment changes equal to or greater than 0.5mm in two contiguous leads, new T-wave inversion equal to or greater than 1mm and/or pseudonormalization of previously inverted T-waves; troponin I equal to or greater than 0.4 ng/mL (which guarantees troponin I levels above the 99th percentile regardless of the kit used); or diagnostic agreement between two physicians.

### Study protocol

At hospital admission for ACS, after signing the informed consent all participants provided baseline information based on standardized questionnaires that included sociodemographic data, cardiovascular risk factors (hypertension, diabetes, obesity, dyslipidemia, smoking, personal or family history of CAD, physical inactivity, cocaine use and menopause) and the use of previous medication. Clinical conditions were self-reported.

Three physicians were independently responsible for reviewing information and validating ACS cases. The study protocol also included blood sample collection for laboratory measurements, including troponin I, MB-creatine kinase, complete blood count and lipid profile (including total cholesterol, HDL-C, LDL-C, and triglycerides).

After 30 days of the acute event, all participants were invited to update their information about cardiovascular risks factors. At six months and annually after the initial event, patients were contacted by phone to update their information, their vital status, cardiovascular history, and medication use. Whenever a patient reported a new potential ACS event, an investigation was initiated to obtain further information. More details about ERICO were described elsewhere.^10^

### Outcomes

Information on all-cause, CVD and CAD mortality were obtained from the ERICO study. Vital status was updated trough medical records and death certificates. Mortality data were confirmed by death certificates obtained from São Paulo’s statistics system (PRO-AIM, Program for Improvement of Mortality Information in the Municipality of São Paulo), São Paulo state health offices (SEADE foundation, Sao Paulo state Health Data Analysis System) and the Brazilian Ministry of Health. On a regular basis, the research team prepared a list of individuals who were reported as dead or with whom contact had been lost. State and municipal health agencies searched their databases for death certificates and reported the results to the ERICO study research team. In the present study, the basic cause of death was used. Two physicians independently analyzed the death certificate and, when necessary, we reclassified the underlying cause of death. Any disagreement between them was resolved through discussion with a third reviewer. Cardiovascular cause of death was defined as any cardiovascular diagnosis classified as “Diseases of the circulatory system” of the International Classification of Diseases version 10 (ICD-10), chapter IX or as ICD-10 code R57.0 “Cardiogenic Shock”. Each event was classified using predefined international criteria.^13 , 14^Participants’ mortality was classified as “post-myocardial infarction mortality” whenever fatal CAD was identified as the main cause of death. For this purpose, the definition of myocardial infarction (ICD-10 code I21.X) was used. All-cause mortality referred to death, regardless of underlying cause recorded.

The study protocol was approved by the Institutional Review Board addressing research in human beings. All subjects provided a written informed consent for the study.

### Statistical analysis

Descriptive statistics of participants are presented by therapeutic strategy (exclusive medical therapy as the reference group, PCI, and CABG). Categorical variables, presented in absolute and relative frequencies, were analyzed using the chi-square test. As no parametric distribution was observed by the Kolmogorov-Smirnov normality test, continuous variables are presented as median values with respective interquartile range (IQR) and compared between therapeutic strategies using the Kruskal-Wallis test.

We performed survival analyses by Kaplan-Meier curves^15^and Cox proportional hazards models (hazard ratios [HR] with respective 95% confidence intervals [CI])^16^to evaluate cumulative all-cause, CVD and CAD mortality rates according to the therapeutic strategies (exclusive medical therapy as the reference group, PCI and CABG). Patients were followed-up for seven years, with a median of 1,460 days (four years). Therefore, Cox regression and hazard ratio analyses were conducted in 180 days and yearly up to four years after an acute event. We calculated the Cox regression models as follows: crude, age-sex adjusted and further adjusted for history of previous CAD, ACS subtype (UA, NSTEMI, STEMI), smoking (past, current and never), hypertension (y/n) diabetes (y/n), dyslipidemia (y/n), ejection fraction, and according to the number of obstructed (≥ 50%) major coronary arteries or any of its major branches (anterior descending artery, circumflex artery and right coronary artery) – no obstruction (all vessels had obstruction <50%), one-vessel (obstruction ≥50% in one major coronary artery or any of its major branches), two-vessel (obstruction ≥50% in two major coronary arteries or its major branches) and multivessel-disease [obstruction in all three major coronary (or its major branches) or in the left main coronary artery (LMCA) or presence of previous CABG].

We also restricted our analyses to NSTEMI cases to better clarify the association between long-term mortality and therapeutic intervention. All tests were two-tailed with a significance of <0.05. Statistical analyses were performed using SPSS® Statistics version 25.0 from IBM®.

## Results

### Clinical and sociodemographic characteristics

Baseline characteristics of ERICO participants by therapeutic strategies after ACS are shown in Table 1 . Overall, mean age was 62±12.9 years, most participants were men 493 (61.6%), white 536 (67.2%), married 506 (63.5%) and had low education level (62,8% had elementary school education). Median time between the onset of ACS symptoms and the first intervention (PCI or CABG) was four days (IQR: 1-8 days) during hospitalization at HU-USP (median: 8 days, IQR: 4-13 days), without significant difference between those who underwent PCI and CABG.

**Table 1:** – Baseline characteristics of the 800 participants of the Strategy of Registry of Acute Coronary Syndrome ERICO cohort according to therapeutic strategies

Sociodemographic Characteristics	MT (n=343)	PCI (n=400)	CABG (n=57)	Total (n=800)	p-value
Median age, years (IQR)	62 (54-71)	61 (52-70)	65 (58-74)	61 (53-71)	0.04
Men (%)	198 (57.7)	259 (64.8)	36 (63.2)	493 (61.6)	0.14
**Race (%)**					0.63
White	222 (64.7)	273 (68.6)	41 (71.9)	536 (67.2)	
Brown	99 (28.9)	103 (25.9)	11 (19.3)	213 (26.7)	
Black	20 (5.8)	18 (4.5)	4 (7)	42 (5.3)	
Asian	2 (0.6)	4 (1)	1 (1.8)	7 (0.9)	
**Marital status (%)**					0.20
Single	40 (11.8)	53 (13.3)	7 (12.3)	100 (12.5)	
Married	204 (60)	267 (66.8)	35 (61.4)	506 (63.5)	
Divorced	32 (9.4)	32 (8)	7 (12.3)	71 (8.9)	
Widowed	64 (18.8)	48 (12)	8 (14)	120 (15.1)	
**Education (%)**					0.65
No formal education	47 (13.7)	43 (10.8)	6 (10.5)	96 (12)	
Elementary	214 (62.4)	248 (62)	40 (70.2)	502 (62.8)	
High-school	57 (16.6)	71 (17.8)	8 (14)	136 (17)	
College	25 (7.3)	38 (9.5)	3 (5.3)	66 (8.3)	
**Cardiovascular risk factors**					
Smoking (%)					<0.001
Current	78 (23.8)	141 (36.3)	9 (17.0)	228 (29.6)	
Past	133 (40.5)	148 (38.1)	25 (47.2)	306 (39.8)	
Never	117 (35.7)	99 (25.5)	19 (35.8)	235 (30.6)	
Median BMI, kg/m^2^(IQR)	27 (24-30)	27 (24-30)	26 (25-30)	27 (24-30)	0.55
Obesity (%)	79 (24.3)	99 (25.9)	11 (23.9)	189 (25.1)	0.87
Hypertension (%)	256 (75.5)	286 (72.8)	46 (80.7)	588 (74.5)	0.38
Diabetes (%)	139 (41.1)	140 (36.2)	28 (50)	307 (39.3)	0.09
Dyslipidemia (%)	155 (51)	197 (55.2)	33 (64.7)	385 (54.1)	0.16
Sedentarism (%)	222 (69.2)	256 (67.4)	43 (78.2)	521 (68.9)	0.27
*Total cholesterol, mg/dl	172 (141-208)	162 (134-204)	180 (148-212)	177 (148-210)	0.02
*LDL cholesterol, mg/dl	103 (78-132)	96 (75-131)	108 (81-134)	108 (80-126)	0.04
HDL cholesterol, mg/dl	37 (31-44)	37 (31-44)	37 (31-43)	38 (32-50)	0.51
Triglycerides, mg/dl	133 (98-189)	123 (94-176)	147 (106-202)	123 (90-162)	0.04
**Types ACS (%)**					<0.0001
Angina	108 (31.5)	69 (17.3)	17 (29.8)	194 (24.3)	
NSTEMI	160 (46.6)	159 (39.8)	32 (56.1)	351 (43.9)	
STEMI	75 (21.9)	172 (43)	8 (14)	255 (31.9)	
Previous CAD (%)	88 (27.8)	80 (21.2)	14 (25.5)	182 (24.3)	0.13
LVEF	58 (43-67)	58 (45-67)	60 (44-65)	58 (45-67)	0.34
*Heart failure (%)	69 (24.0)	55 (15.5)	2 (4.5)	126 (18.3)	0.001

* All these comorbidities were self-reported by participants based on their medical diagnosis and treatment. MT: conservative medical therapy; PCI: percutaneous coronary intervention; CABG: Coronary artery bypass grafting; BMI: Body mass index; CAD: coronary artery disease; NSTEMI: non-ST-elevation myocardial infarction; STEMI: ST-elevation myocardial infarction; LVEF: left ventricular ejection fraction. All continuous variables were described as median and interquartile range (IQ) P-values were obtained from chi-Square test or Kruskal-Wallis for categorical and continuous variables, respectively.

Regarding therapeutic strategies, 343 (42.9%) patients underwent exclusive medical therapy (15 had undergone previous chemical thrombolysis), 400 (50.0%) underwent PCI or stent placement (65 had undergone previous chemical thrombolysis), and 57 (7.1%) underwent CABG (one had undergone previous chemical thrombolysis). We observed that participants undergoing CABG were older and had slightly higher levels of total cholesterol, LDL cholesterol and triglycerides. However, higher frequencies of current smokers and STEMI were noticed among individuals who underwent PCI compared to other groups. Although we found a higher frequency of self-reported previous heart failure before the index event among those who underwent exclusive medical therapy, the ejection fraction was not statistically different between treatment groups ( Table 1 ).

Regarding drug therapy at admission, patients who underwent CABG showed the lowest percentage of warfarin use (3.6%; p=0.032) compared to other groups. The highest use of clopidogrel in 30 days was found among patients who underwent PCI (51.5%; p=0.018), and patients who underwent CABG made more frequent use of calcium beta-blocker (20%; p=0.029) compared to other groups. In 180 days, patients who underwent PCI showed highest frequency of clopidogrel use compared to other groups (30%, p=0.014). In one year, we did not find any significant differences regarding medication use (Supplementary Table 1 ).

### Reperfusion therapy versus coronary obstruction

Regarding the extension of the obstructive disease, we found 107 (13.4%) patients with no obstruction, 304 (38.0%) patients with one-vessel-disease, 169 (21.1%) with two-vessel-disease and 220 (27.5%) patients with multivessel disease. Although most patients included in the “exclusive medical therapy” group had mild coronary heart disease, they showed a higher frequency of multivessel disease when compared to the PCI group (28.9% vs. 18.0%, p<0.0001).

Most of the 400 patients undergoing PCI received metal stent (n=325; 75.8%) followed by balloon angioplasty 57 (13.3%) and drug-eluting stent 47 (10.9%). Of note, among patients undergoing PCI, only 40 patients (10%) required a repeated PCI revascularization, and three patients (0.75%) required a CABG after PCI. No patient undergoing CABG needed a new revascularization.

### Mortality and survival rates

Overall, there were 140 deaths post-ACS (88 deaths due to CVD, 52 due to CAD), and the following mortality rates: exclusive medical therapy (76/274; 27.7%), PCI (50/314; 15.9%) and14/48 (29.2%), p=0.001 up to four years of follow-up. The lowest crude survival rates were also detected among individuals who underwent CABG (all-cause and CVD survival p-log rank: 0.001, Figures 1 - 3 [Fig f03001] [Fig f04001] ).

**Figure 1:** – Kaplan-Meier survival curve for all-cause mortality during four years of follow-up; MT: medical treatment; ICP: percutaneous coronary intervention; CABG: coronary artery bypass grafting.

**Figure 2 f03001:** – Kaplan-Meier survival curve for cardiovascular disease mortality during four years of follow-up; MT: medical treatment; PCI: percutaneous coronary intervention; CABG: coronary artery bypass grafting.

**Figure 3 f04001:** – Kaplan-Meier survival curve for myocardial infarction mortality during four years of follow-up; MT: medical treatment; PCI: percutaneous coronary intervention; CABG: coronary artery bypass grafting.

In the Cox regression models, PCI was associated to a lower probability of fatal events during the four-year follow-up: all-cause [multivariate HR: 0.42 (95% CI: 0.26-0.70)] CVD [HR: 0.39 (95% CI: 0.20-0.73)] and CAD [multivariate HR: 0.24 (95% CI: 0.09-0.63)] compared to those submitted to exclusive medical therapy. CABG was correlated to CAD mortality after age and sex adjustment [HR: 2.19 (95% CI: 1.05-4.55)]. However, this risk lost significance in the full model ( Table 2 ).

**Table 2:** – Hazard ratios (HR) [95% Confidence Intervals (95% CI)] of all-cause, cardiovascular and coronary artery disease mortality among ERICO participants according to therapeutic strategies after acute coronary event

All-cause mortality (Total deaths=140)	180 days	1 year	2 years	3 years	4 years
**Crude**					
MT	Reference	Reference	Reference	Reference	Reference
PCI	0.74 (0.41 - 1.35)	0.62 (0.36 - 1.04)	0.53 (0.35 - 0.82)	0.51 (0.34 - 0.75)	0.53 (0.37 - 0.75)
CABG	2.21 (0.99 - 4.93)	2.12 (1.07 - 4.20)	1.32 (0.69 - 2.53)	1.24 (0.68 - 2.25)	1.15 (0.65 - 2.03)
**Age and sex-adjusted**					
MT	Reference	Reference	Reference	Reference	Reference
PCI	0.79 (0.43 - 1.43)	0.64 (0.38 - 1.09)	0.55 (0.36 - 0.85)	0.52 (0.35 - 0.77)	0.55 (0.38 - 0.78)
CABG	2.02 (0.90 - 4.51)	1.91 (0.96 - 3.78)	1.18 (0.62 - 2.26)	1.11 (0.61 - 2.01)	1.04 (0.59 - 1.84)
**Multivariable adjusted^**1**^**					
MT	Reference	Reference	Reference	Reference	Reference
PCI	0.52 (0.22 - 1.25)	0.46 (0.21 - 0.99)	0.38 (0.21 - 0.69)	0.41 (0.24 - 0.7)	0.42 (0.26 - 0.70)
CABG	0.90 (0.25 - 3.21)	1.10 (0.4 - 2.99)	0.59 (0.23 - 1.54)	0.64 (0.27 - 1.54)	0.62 (0.28 - 1.39)
**CVD mortality (Total deaths = 88)**					
**Crude**					
MT	Reference	Reference	Reference	Reference	Reference
PCI	0.81 (0.41 - 1.59)	0.69 (0.37 - 1.3)	0.61 (0.37 - 1.03)	0.50 (0.31 - 0.81)	0.49 (0.31 - 0.78)
CABG	2.61 (1.08 - 6.29)	2.60 (1.2 - 5.65)	1.68 (0.81 - 3.51)	1.43 (0.72 - 2.84)	1.44 (0.75 - 2.77)
**Age and sex-adjusted**					
MT	Reference	Reference	Reference	Reference	Reference
PCI	0.84 (0.43 - 1.67)	0.72 (0.38 - 1.34)	0.63 (0.38 - 1.06)	0.51 (0.32 - 0.83)	0.50 (0.31 - 0.79)
CABG	2.36 (0.98 - 5.69)	2.31 (1.06 - 5.03)	1.49 (0.71 - 3.11)	1.25 (0.63 - 2.49)	1.27 (0.66 - 2.45)
**Multivariable adjusted^**1**^**					
MT	Reference	Reference	Reference	Reference	Reference
PCI	0.56 (0.2 - 1.54)	0.54 (0.22 - 1.32)	0.46 (0.22 - 0.94)	0.40 (0.20 - 0.79)	0.39 (0.20 - 0.73)
CABG	1.15 (0.31 - 4.26)	1.17 (0.37 - 3.66)	0.77 (0.26 - 2.27)	0.83 (0.31 - 2.22)	0.84 (0.34 - 2.07)
**CAD mortality (Total deaths = 52)**					
**Crude**					
MT	Reference	Reference	Reference	Reference	Reference
PCI	0.86 (0.37 - 1.98)	0.62 (0.29 - 1.36)	0.63 (0.32 - 1.23)	0.57 (0.29 - 1.10)	0.51 (0.27 - 0.94)
CABG	3.44 (1.27 - 9.29)	3.38 (1.43 - 7.96)	2.54 (1.12 - 5.76)	2.58 (1.19 - 5.61)	2.42 (1.16 - 5.01)
**Age and sex-adjusted**					
MT	Reference	Reference	Reference	Reference	Reference
PCI	0.91 (0.39 - 2.10)	0.65 (0.30 - 1.41)	0.65 (0.33 - 1.28)	0.59 (0.30 - 1.13)	0.52 (0.28 - 0.97)
CABG	3.16 (1.17 - 8.57)	3.06 (1.3 - 7.25)	2.29 (1.00 - 5.2)	2.31 (1.06 - 5.03)	2.19 (1.05 - 4.55)
**Multivariable adjusted^**1**^**					
MT	Reference	Reference	Reference	Reference	Reference
PCI	0.49 (0.12 - 2.02)	0.39 (0.10 - 1.50)	0.31 (0.10 - 0.91)	0.29 (0.10 - 0.84)	0.24 (0.09 - 0.63)
CABG	0.85 (0.17 - 4.21)	1.01 (0.26 - 3.93)	0.76 (0.21 - 2.75)	0.91 (0.29 - 2.86)	0.93 (0.33 - 2.58)

*^1^Adjusted for: age and gender, type of ACS (unstable angina, ST-elevation myocardial infarction, non-ST-elevation myocardial infarction), history of previous CAD, smoking (past, current and never), hypertension, (y/n), diabetes (y/n), dyslipidemia (y/n), ejection fraction and according to the number of obstructed (≥ 50%) major coronary arteries or any of its major branches (no obstruction, 1-vessel-disease, 2-vessel-disease and multivessel-disease); MT: conservative medical therapy; PCI: percutaneous coronary intervention; CABG: coronary artery bypass grafting.*

We chose patients who underwent exclusive medical therapy as the reference group because there were fewer individuals with severe forms of ACS [angina n=108 (31.5%)] and the highest number of patients with no obstruction 103 (96.3%).

Additional adjustments for medication use such as aspirin, lipid lowering drugs, angiotensin converting enzyme inhibitors, β-blocker, or warfarin at baseline and after the acute event did not modify our findings. For PCI, four-year mortality HRs were all-cause 0.39 (95% CI: 0.25-0.61), CVD 0.36 (95% CI: 0.20-0.65) and CAD 0.31 (95% CI: 0.14-0.70). For CABG, the following mortality HRs: all-cause 0.91 (95% CI: 0.47-1.76), CVD 1.06 (95% CI: 0.49-2.32) and CAD 1.27 (95% CI: 0.53-3.05).

Restricting our analyses to the 351 NSTEMI cases (n=80 deaths), we found similar results regarding the inverse association with long-term mortality risks and PCI intervention compared to those patients under exclusive medical therapy (Supplementary Table 2 ). In fact, CVD and CAD HRs in our study were even lower for NSTEMI patients submitted to PCI (four-year risk: CVD 0.28 [95% CI: 0.12-0.67] and CAD 0.13 [95% CI: 0.03-0.63]) compared to the same mortality risks observed when we included all sample in the Cox model.

## Discussion

### Main findings of acute coronary event and mortality

In our sample, the PCI group was the only one significantly associated with lower all-cause, CVD and CAD mortality during the four-year follow-up ( Central Illustration ).

**Central Illustration:** : Prognosis Related to Reperfusion Therapy Post-Acute Coronary Syndrome in Secondary Care: Long-Term Survival Analysis in the ERICO Study

Some studies that evaluated therapeutic interventions after an ACS event restricted their samples to NSTEMI patients and found no significant association comparing invasive strategies versus more conservative strategies.^5 - 7^

In a randomized clinical trial (RCT) by Boden et al.,^5^920 NSTEMI patients were randomized into two groups (invasive or conservative treatment), with no significant differences reported for all-cause mortality during 23 months of follow-up. McCullough et al.^6^investigated 201 patients in a RCT, with suspected ACS who were ineligible for thrombolysis and no differences between treatment groups were found during 21 months of follow-up.^6^Another RCT with 313 NSTEMI patients allocated in two groups – early aggressive or an initially conservative strategy – also found no significant differences regarding mortality during 12 months of follow-up.^7^

In our study, when we restricted our sample to NSTEMI patients, those subjected to PCI showed lower all-cause, CVD and CAD mortality when compared to those who underwent medical treatment alone. Of note, more than 50% of deaths in the total sample occurred among NSTEMI cases (80/140 total deaths); thus, this subgroup was the one that most benefited from PCI. And differently from previous studies,^5 , 6^we considered a longer follow-up that might allowed us to detect a beneficial effect of PCI in all-cause, CVD and CAD mortality for all ACS subtypes.

ERICO patients were first seen in a non-tertiary setting, thus the interval between the onset of ACS symptoms and cardiac intervention in most cases was longer (after 24-48 hours) than in studies conducted in specialized centers, where cardiologic procedures are usually performed within 24- 48 hours after an acute event.^5 - 7^Even considering this limitation, our main finding favors PCI for NSTEMI cases. Further, previous chemical thrombolysis performed in the acute phase in 16.3% of PCI subgroup might also have contributed to a better long-term prognosis in our study, even with the highest frequencies of STEMI and current smokers in this subgroup, compared to other subgroups (CAGB and exclusive medical therapy).

Another possible explanation for this long-term protective effect observed in the PCI subgroup is the higher frequency of bare metal stent (75.8% of PCI cases). In fact, with the advent of bare metal stent the need for restenosis has become less frequent.^17 , 18^Recent studies have shown a lower rate of revascularization in patients receiving bare stent metal (19.8%) and drug-eluting stents (16.5%) [HR: 0.76 (95% CI: 0.69-0.85) p<0.001], but no significant differences in all-cause mortality or non-fatal myocardial infarction during a six-year follow-up. In our study, the rate of revascularization after the first PCI was even lower (10%).

Our findings differ from those in the literature, which may be explained by the fact that our sample included all types of ACSs, and not only high-risk patients with multivessel disease. But even considering NSTEMI cases, our results were confirmed. Moreover, our findings capture a period witnessing three different stent eras (balloon angioplasty, bare metal, and drug-eluting stents), which might explain why our results favor PCI as an early invasive therapy, related to better long-term survival in this low-income population.

There is only one study showing significant associations between ACS and mortality with significant associations. Wijeysundera et al.^8^evaluated 50,302 NSTEMI patients according to reperfusion treatment seven days after their index angiogram: 68.2% underwent revascularization [PCI (n=28,011) and CABG (n=6,227)] or exclusive medical therapy (n=16,014). During the follow-up of six years, patients undergoing CABG [HR: 0.53 (95% CI: 0.47–0.60)] and PCI [HR: 0.64 (95% CI: 0.60–0.69)] had lower mortality risks compared to those undergoing exclusive medical therapyy.

In our study, patients who underwent CABG (n=52), after the index event, had the lowest survival rates for CAD mortality in four years of follow-up in crude and age and sex adjusted models. However, these findings were not confirmed in the multivariate models. Probably the low number of CABG cases did not allow us to find statistical significance in our multivariate analysis for all-cause, CVD and CAD mortality, as we observed for the PCI subgroup.

### Strengths

This study provides a single opportunity to evaluate long-term survival and mortality (all-cause, CVD and CAD) according to therapeutic strategies adopted post-ACS in a low-middle income population seen in secondary care. Moreover, most previous studies did not describe the effects of clinical treatment on specific mortality, as we did.

### Limitations

Since this is an observational study, we cannot extrapolate our findings to other populations. In addition, there are confounders that cannot be controlled, including selection bias of the therapeutic strategies. Besides that, invasive angiography for the diagnosis of coronary obstruction was not performed by a single or a restricted team of professionals. However, a cardiologist from the ERICO study revised all cases carefully and performed the classification according to the extension of the obstructive disease.

Finally, although we observed a low rate of cardiologic reintervention in the subgroups (less than 1% of participants required CABG after PCI) in the four-year follow-up, the possibility that higher rates may be found in a longer follow-up cannot be excluded.

## Conclusions

In the ERICO study, PCI reperfusion strategy was associated to better long-term prognosis in all ACS subtypes during four-year follow-up. These findings were confirmed even considering only NSTEMI cases. Advances in technology with the emergence of bare metal stent and drug-eluting stents might have favored PCI as treatment of choice in this low-middle income population.

## * Supplemental Materials

For additional information, please click here .
